# Autophagy-Related Deubiquitinating Enzymes Involved in Health and Disease

**DOI:** 10.3390/cells4040596

**Published:** 2015-10-05

**Authors:** Fouzi El Magraoui, Christina Reidick, Hemut E. Meyer, Harald W. Platta

**Affiliations:** 1 Biomedizinische Forschung, Human Brain Proteomics II, Leibniz-Institut für Analytische Wissenschaften - ISAS -e.V. 44139 Dortmund, Germany; E-Mails: fouzi.elmagraoui@isas.de (F.E.); helmut.e.meyer@isas.de (H.E.M); 2 Biochemie Intrazellulärer Transportprozesse, Ruhr-Universität Bochum, 44801 Bochum, Germany; E-Mail: christina.reidick@rub.de

**Keywords:** deubiquitination, ubiquitination, DUB, USP, autophagy, mitophagy, cancer, neurodegeneration

## Abstract

Autophagy is an evolutionarily-conserved process that delivers diverse cytoplasmic components to the lysosomal compartment for either recycling or degradation. This involves the removal of protein aggregates, the turnover of organelles, as well as the elimination of intracellular pathogens. In this situation, when only specific cargoes should be targeted to the lysosome, the potential targets can be selectively marked by the attachment of ubiquitin in order to be recognized by autophagy-receptors. Ubiquitination plays a central role in this process, because it regulates early signaling events during the induction of autophagy and is also used as a degradation-tag on the potential autophagic cargo protein. Here, we review how the ubiquitin-dependent steps of autophagy are balanced or counteracted by deubiquitination events. Moreover, we highlight the functional role of the corresponding deubiquitinating enzymes and discuss how they might be involved in the occurrence of cancer, neurodegenerative diseases or infection with pathogenic bacteria.

## 1. Introduction

A central building block of cellular physiology is the intracellular trafficking of macromolecules. This involves the transport and distribution of biogenetic cargoes that are required for the formation and maintenance of cellular compartments, but also concerns the regulated removal of aberrant macromolecules, organelles or endocytosed extracellular factors. The hydrolytic breakdown takes place in the lysosome [1]. The cargo is delivered to the lysosome by vesicular carriers via different mechanisms, which involve membrane involution processes, such as endocytosis for extracellular factors [2], autophagy for intracellular cargo [3] or phagocytosis and xenophagy in the case of pathogen removal [4]. These processes are regulated via the posttranslational modification of cargoes and regulatory key factors by ubiquitination [5]. In this review, we will focus on the functional role of the dynamics underlying ubiquitination and deubiquitination of autophagy-related proteins and cargoes.

### 1.1. Autophagy

The term “autophagy” is derived from Greek and means “self-eating”. The main physiologic role of autophagy is thought to be autodigestion of cellular components. This is especially required under conditions that lead to starvation of the cells, because the recycled nutrients can be utilized by the cell again. Moreover, aberrant organelles, pathogenic protein aggregates or intracellular bacteria can be degraded by autophagy [6].

In general, several types of autophagic pathways can be defined. The term macroautophagy is mostly used when large portions of cytosolic components are delivered to the lysosome in a process that is also called bulk autophagy [7]. When the cell is exposed to starvation conditions, the cellular components are engulfed by autophagic membranes. These structures are referred to as autophagosomes, which then are delivered to the lysosome for their breakdown [8]. In certain cases, the autophagosome fuses first with endosomes, which carry cargo derived from the plasma membrane, in order to form amphisomes, before the fusion step with the lysosome occurs [8,9]. Therefore, autophagy promotes the disposal of aberrant cellular components and enables the reuse of the macromolecules, like amino acids, gained by this autodigestion process.

Under conditions when only certain components should be degraded, potential cargoes can be specifically marked. The selectivity can be defined by cargo-specific autophagy-receptors [10,11] or arginylation of the target [12]. In mammalian cells, many targets are modified by ubiquitin [5]. The priming via ubiquitin enables the selective recognition by autophagy receptors, which bind the attached ubiquitin, as well as key factors of the autophagy machinery, such as LC3 (light chain 3) or PtdIns3P (phosphatidylinositol 3-phosphate) [13]. Therefore, the autophagy receptors connect the cargo to the assembly of the autophagy machinery and, finally, the formation of the autophagosome, which engulfs the cargo [14].

In general, the autophagy machinery consists of ATG (autophagy-related gene) proteins, which are required for the formation of the autophagosome. ATG proteins can be structurally and functionally divided into several subgroups. Important examples concern early signaling complexes, like the ULK1(Atg1)-complex, which is assembled around the protein kinase ULK1 [15,16], or the so-called PI3K-III (class III phosphatidylinositol 3-kinase) complex, which contains the multivalent adaptor protein Beclin 1(Atg6) and the PtdIns3P-generating lipid kinase VPS34 [17,18]. Moreover, two ubiquitin-like conjugation cascades are involved, namely the ATG12-conjugation system, as well as the LC3(Atg8)-conjugation system. Especially the modification of the ubiquitin-like molecule LC3 with phosphatidylethanolamine represents a hallmark of autophagy induction [19,20].

Apart from the described autophagosome-based variants of autophagy, namely the bulk autophagy and selective autophagic pathways, two other basic forms of autophagy have been described. The first one is assigned as microautophagy, which requires the engulfment of cargo mainly by lysosomal membranes [21]. The second one is called chaperone-mediated autophagy, because it is based on the concept that the chaperone-bound cargo is directly targeted to the lysosome without additional vesicular carriers [22].

In general, autophagy represents an evolutionarily-conserved process that is integral for cellular homeostasis and the response to different stress conditions. Therefore, defects in autophagy pathways are often associated with diverse human pathologies, including infectious diseases [23], neurodegenerative disorders [24,25] and cancer [26]. It is important to note that autophagy has been referred to as a “double-edged” sword in the context of cancer. Dysfunctional autophagy can contribute to tumorigenesis and the occurrence of cancer, while the still functional autophagy of tumor cells that have already transformed via another mechanism protects and supports the tumor [27,28].

All of the mentioned topics of autophagy have been covered by excellent recent reviews (see the publications cited above). Here, we describe the regulation of autophagic signaling, as well as selective autophagy pathways by the modulation of ubiquitination dynamics. While previous reviews have focused on the attachment of ubiquitin to autophagy-related proteins and cargoes [5,29,30], this manuscript will discuss the growing body of evidence concerning the functional role of deubiquitination events in autophagy.

### 1.2. Deubiquitinating Enzymes

The posttranslational modification of proteins by ubiquitination is highly conserved in eukaryotic cells. It requires the concerted interplay of different enzyme classes. While the ubiquitin activation enzyme (E1) starts the process in an ATP-dependent manner, the ubiquitin conjugation enzyme (E2) takes over the activated ubiquitin and cooperates with a ubiquitin-protein isopeptide ligase (E3). The E3 enzyme can interact in a specific way with the substrate and, therefore, enables modification of the target protein by mono- or poly-ubiquitination. Depending on its modification pattern, the target protein can be degraded by the proteasome, translocated to a new compartment or activated for the association with new binding partners [31,32,33]. However, when the ubiquitin-dependent event is completed, the ubiquitin-signal itself has to be removed. This process is catalyzed by a large group of ubiquitin-cleaving proteases, the deubiquitinating enzymes (DUBs), which are also called deubiquitinases, ubiquitin proteases and ubiquitin-hydrolases. DUBs are involved in the editing or removal of ubiquitin signals in order to alter or terminate the biological function of a protein. DUBs can be subdivided into two main families: the cysteine proteases and the metalloproteases. While the cysteine protease superfamily of DUBs includes several classes, like the ubiquitin C-terminal hydrolases (UCH), the ubiquitin-specific protease (USP/UBP), the ovarian tumor (OTU) and the Machado–Josephin (MJD) protease, the DUBs of the metalloprotease type consist of only one class, the Jab1/Mov34/Mpr1 Pad1 (MPN+) (JAMM) superfamily [34].

Even though the function of the DUBs is highly conserved, they display a distinct structural organization. While UCH-type DUBs contain a core domain structure that adopts a catalytic triad-geometry closely resembling Papain [35], the USP-type DUBs consist of three sub-domains, namely a finger, a palm and a thumb, which form a “right hand” [36,37]. The OTU-core domain is classified by five β-strands sandwiches between helical domains [38,39,40,41]; the MJD structure matches the UCH domain structure, while the Jab1/Mov34/Mpr1 Pad1 (MPN+) (JAMM) superfamily differs in structure and principal function. The metalloproteases coordinate zinc ions with histidine, aspartate and serine residues, which allow water molecules to attack the ubiquitin-substrate isopeptide bond [42].

The functional contexts in which DUBs play a role are as diverse as the role of ubiquitin itself. Along with the regulation of protein targeting, protein stability and quality control, as well as signaling events [43,44], they are also involved in the homeostasis of organelles. While some contribute to the biogenesis of certain organelles [45,46,47], others are involved in their degradation via autophagy (see Section 2).

## 2. Deubiquitinating Enzymes Involved in Autophagy

Several examples show that deubiquitination events can regulate autophagy. The general effect of DUB inhibitors has been reported [48,49,50], and the involvement of certain DUBs, like USP22 [51] and UCH-L1 [52,53,54,55], has been demonstrated. However, the specific molecular target is known in relatively few cases. Interestingly, certain deubiquitinases influence different types of autophagic pathways. Conversely, several different DUBs can be involved in the same type of autophagic pathway, because they can act at different steps of this process and target distinct substrate proteins. Because alterations in the functionality of autophagy are often linked to the occurrence of several diseases, the activity of the autophagy-related deubiquitinases is often associated with the occurrence of disease.

### 2.1. A20

The deubiquitinase A20, which is also called TNFAIP3 (tumor necrosis factor, α-induced protein 3), belongs to the OTU-family [41]. It has been described as a negative regulator of NF-κB [56]. The functional inactivation of A20 is often associated with different types of cancers, like diffuse large B-cell lymphoma (DLBCL) [57], Hodgkin’s lymphoma [58] or multiple myeloma of the bone marrow [59]. The reintroduction of functional A20 into an A20-deficient cell line leads to a suppression of cell growth, the reduction of NF-κB target gene expression and the induction of apoptosis. These results demonstrate that A20 can act as a tumor suppressor in the analyzed lymphomas [58,59,60]. Furthermore, A20 has been identified as a susceptibility gene also for multiple inflammatory diseases, including systemic lupus erythematodes, diabetes mellitus type 1 and colitis [61,62,63]. Moreover, A20 knock-out mice show a higher rate of spontaneous inflammations, which supports the assumption that a reduction of A20 expression is associated with the development of autoimmune disease [64].

Moreover, a role of A20 in the early events of xenophagy, the autophagy-related transport and lysosomal degradation of pathogens, has been described in macrophages [65]. Macrophages exhibit Toll-like receptors (TLRs) on their plasma membrane. TLRs have a central function in innate immunity, as well as adaptive immunity, because they are required for the recognition of the conserved pathogen-associated molecular patterns (PAMPs). The recognition of bacterial lipopolysaccharides by TLR4 induces a signaling cascade that triggers the phagocytosis of the pathogen, as well as the induction of autophagy [66,67]. The intracellular TLR4-adaptor proteins MyD88 (myeloid differentiation primary response gene 88) and TRIF (TIR-domain-containing adapter-inducing interferon-β) are capable of attracting the PI3K-III signaling complex component Beclin 1 and contribute to its activation when TLR4 binds LPS molecules [68].

The dissociation of the inhibitory factor Bcl-2 from Beclin 1 is also regulated by ubiquitination and deubiquitination events, which involve the RING-type E3 ligase TRAF6 (tumor necrosis factor receptor (TNFR)-associated factor 6) and the deubiquitinase A20, respectively [65].

**Figure 1:**
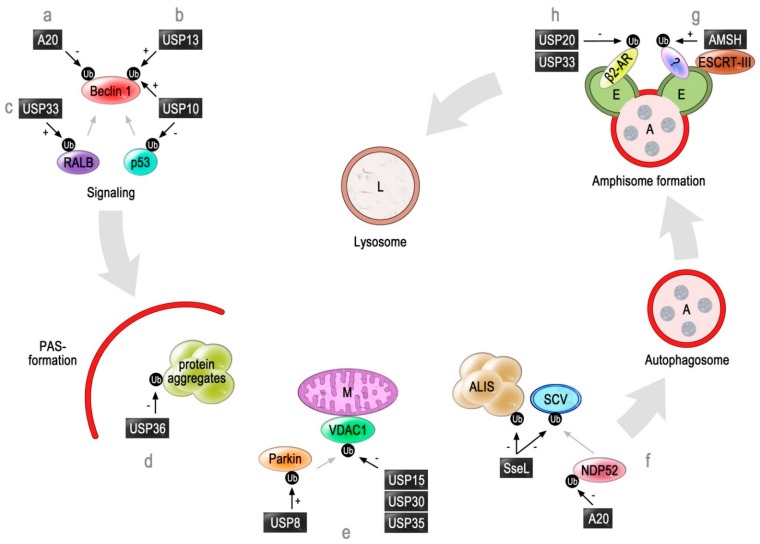
Functional role of deubiquitinating enzymes in autophagy. An overview of the involvement of deubiquitinases in different steps of the autophagy pathway. In general, the stimuli that elicit autophagy induction can be integrated by certain signaling cascades. This results in the formation of the pre-autophagosomal structure (PAS), which is a newly-formed membrane that surrounds the potential cargo, like cytosolic components, protein aggregates or damaged organelles. The formed compartment is called the autophagosome. In certain cases, it can fuse with endosomes to generate an amphisome. Finally, the amphisome fuses with the lysosome, and the cargo is degraded. Depicted is a composite figure, which displays an overview of the function of the DUBs related to autophagy. Therefore, not all of the shown enzymes may be active in the same autophagic pathway. (**a**) A central event in early autophagic signaling is the activation of Beclin 1 and, thereby, the formation of PtdIns3P by the Beclin 1-containing PI3K-III complex. In macrophages, Beclin 1 is polyubiquitinated with Lys63-linked ubiquitin-chains, which supports xenophagy. A20 deubiquitinates Beclin 1 and, thereby, inhibits the autophagic degradation of bacteria. (**b**) USP10 and USP13 deubiquitinate polyubiquitinated Beclin 1 and thereby contribute to its stabilization. Interestingly enough, USP10 also deubiquitinates p53, thereby eliciting a balanced breakdown of Beclin 1 in order to prevent the uncontrolled induction of autophagy. (**c**) USP33 deubiquitinates mono-ubiquitinated RALB. This allows RALB to associate with Beclin 1-containing complexes and to induce autophagy. (**d**) Selected cargoes of autophagy are engulfed by the PAS in order to form autophagosomes (A). Insoluble proteins form aggregates. The aggregates are marked with ubiquitin in order to be recognized by autophagy-receptors, like p62, in a process called aggrephagy. USP36 counteracts this process by removing the chains from the substrate. (**e**) Mitochondria (M) are degraded via mitophagy. In the case of Parkin-dependent mitophagy, the ubiquitination of mitochondrial outer membrane proteins, for example VDAC1, by the E3 ligase Parkin is required. USP8 keeps Parkin in its active form, as it prevents Lys6-linked auto-ubiquitination. In contrast, USP15, USP30 and USP35 inhibit mitophagy by removing the ubiquitin-signal from Parkin-targets. (**f**) Intracellular *Salmonella* species are usually surrounded by a protective membrane (SCV, *Salmonella* containing vacuole). However, the penetration of the cell also causes stress-induced protein aggregates (aggresome-like induced structures (ALIS)). Damaged SCVs, as well as ALIS are ubiquitinated and then recognized by the autophagy-receptor NDP52. NDP52 itself has to be activated by the E3 ligase TRAF6 via the attachment of Lys63-linked polyubiquitin chains. A20 counteracts the function of NDP52 in order to prevent an overactivation. The DUB SseL is secreted by *Salmonella* in order to deubiquitinate ALIS and SCVs with the aim to hide the presence of the bacterium from the autophagy-machinery. (**g**) In several cases, the autophagosome fuses with late endosomes (E) to form amphisomes. This fusion requires a functional ESCRT-III machinery, as well as the deubiquitinase AMSH. Moreover, USP20 and USP33 are negative regulators of endocytosed β2-AR before the fusion step with the amphisome. Finally, the autophagic cargo is delivered to the lysosome to form an autolysosome for degradation. Legend: “Ub” = ubiquitin (this can involve different kinds of ubiquitination); “black arrows” = target of a DUB, which can support autophagy (“+”) or inhibit autophagy (“−”); “grey arrow” functional interaction

TRAF6 catalyzes the formation of a Lys63-linked polyubiquitin chain within the BH3 domain of Beclin 1 [65]. In general, Lys63-linked ubiquitin chains have almost always a non-proteolytic function in protein targeting [69]. Because Bcl-2 is not able to bind to the modified BH3 domain anymore, Beclin 1 can induce autophagy [65,70]. However, this TRAF6-dependent polyubiquitination of Beclin 1 is antagonized by the deubiquitinase A20 [65] (Figure 1a). A20 specifically hydrolyzes the peptide-bond between ubiquitin and Beclin 1. This again makes the interaction of Bcl-2 with the BH3-domain of Beclin 1 possible and therefore antagonizes the PtdIns3P-dependent induction of autophagy.

TRAF6 is not the only E3 enzyme involved in the ubiquitination of Beclin 1, because also the HECT-type ligase Nedd4 and the Cullin-type ligase complex Rbx1/Cul4/DDB1/Ambra1 can modify Beclin 1 [71,72]. However, the putative corresponding DUBs that are possibly involved in the other functions of Beclin 1 are not known. Conversely, it remains to be elucidated if A20 is involved in the deubiquitination of other TRAF6-substrates, like the kinases UKL1 [73] and TAK1 (transforming growth factor β-activated kinase 1) [74,75].

The regulatory impact of A20 on autophagic processes seems to have different layers of complexity. Even though overexpression of A20 counteracts the TRAF6- and Beclin 1-dependent autophagy upon TLR4-activation [65], the principle presence of A20 is required for this process [76]. Similarly to Beclin 1, NDP52 (nuclear dot protein 52 kDa) is ubiquitinated by TRAF6 in order to be activated. NDP2 is an autophagy-receptor for ubiquitin-decorated cytosolic bacteria [77] (Figure 1f). This process is reversed by A20, which deubiquitinates and thereby inactivates NDP52 [76]. However, downregulation of A20 results in an enhanced activation of NDP52. This results in a faster lysosomal-degradation of selected TLR4-adaptors (MyD88, TRIF), as well as of TRAF6, which shuts down TLR-signaling [76]. Therefore, a balanced activity of A20 is required for the correct context-dependent modulation of the ubiquitin chains on NDP52.

It is interesting to note that A20 itself can also become a target of autophagy under certain conditions. F4/80(hi) macrophages of the spleen, peritoneum and kidney can sequester A20 depending on the autophagy-receptor p62 [78]. This results in a temporarily enhanced NF-κB signaling, which enables the macrophages to secrete chemokines and to recruit neutrophils [78].

These studies demonstrate that A20 is involved in different aspects and sometimes even partially opposing regulatory steps in early autophagy, indicating the possible role in separate cargo-selective pathways.

### 2.2. USP10 and USP13

The involvement of the deubiquitinases USP10 and USP13 in autophagic processes has been discovered as a result of a systematic screen for small chemical compounds. The aim was to discover inhibitors of autophagy in mouse embryo fibroblasts (MEFs). The identified substance, Spautin-1 (specific and potent autophagy inhibitor-1), was shown to block macroautophagy via the inhibition of the activity of USP10 and USP13 [79]. The functional impairment of these deubiquitinases caused polyubiquitination and degradation of several PI3K-III components, such as VPS34, Beclin 1, VPS15 and ATG14L [79]. This resulted in a reduced PtdIns3P production by the PI3K-III complex, which finally hampered autophagy at an early stage [79] (Figure 1b). Because a direct interaction of USP13 with Beclin 1 has been detected, it is thought that USP13 counteracts the ubiquitination of Beclin 1 by yet unknown ligases [79]. Currently, it is not known if USP13 and USP10 also protect the other PI3K-III complex components directly or, alternatively, indirectly via the stabilization of Beclin 1. This second assumption is based on the reports from yeast [80], as well as mammalian cells [81] showing that a deletion of yeast Atg6 or siRNA-mediated knock-down of Beclin 1, respectively, trigger the instability of other PI3K-III complex subunits.

The close functional interconnection between the central PI3K-III subunits and the DUBs is demonstrated by the interesting observation that knock-down of VPS34 and Beclin 1 causes instability of USP10 and USP13, which strongly indicates the existence of a regulatory feedback loop [79].

In the context of anti-cancer treatment studies, it is interesting to note that Spautin-1 supports the treatment of chronic myeloid leukemia and ovarian cancer cells, when combined with other drugs [82,83].

Spautin-1 is a rare case of a DUB-inhibitor that apparently targets specific deubiquitinases, namely USP10 and USP13, and whose basic effect on autophagy has been elucidated. However, given that the PI3K-III complex has also a functional role in endocytic transport and cytokinesis [81], it will be of interest if the USP10/USP13/Beclin 1-axis is also relevant for these processes.

Furthermore, the finding that USP10 can be co-isolated with ULK1 [84] suggests another hypothetical connection to autophagic processes. Because ULK1 phosphorylates the mTOR-inactivator AMPK (AMP-activated protein kinase) [85], as well as the PI3K-III constituents Ambra 1 [73,86] and Beclin 1 [87,88], it plays an essential role during the early events of autophagy induction. However, a direct deubiquitination of ULK1 by USP10 remains to be demonstrated.

Another relevant link of USP10 to autophagy is its role as a deubiquitinating enzyme for p53 [89]. It was shown that USP10 can stabilize cytosolic p53. However, this study also demonstrates that USP10 does not discriminate between wildtype or mutated p53 and, thereby, either suppresses or supports tumorigenesis depending on the p53 species present [89,90].

Recently, the newly-identified interaction of p53 with Beclin 1 is thought to regulate the cellular decision between the induction of apoptosis or, alternatively, autophagy in embryonal carcinoma cells [91]. On the p53 side, this balance is held up by the p53-elicited expression of E3 ligases that ubiquitinate and thereby destabilize Beclin 1 [91] and VPS34 [92], which prevents autophagy induction. On the Beclin 1 side, the deubiquitinase USP10 is stabilized by Beclin 1, which in turn contributes to p53 stabilization [79] and protection against degradation by Mdm2 or another E3 enzyme [93]. This mechanism might produce a balanced, constitutive level of the key regulatory factors p53 and Beclin 1.

In summary, the example of USP13 and USP10 demonstrates the possibilities that a DUB-specific inhibitor can offer. Moreover, especially the case of USP10 illustrates the complex interplay and interdependency of autophagy and apoptosis based on factors with overlapping functions, like DUBs.

### 2.3. USP33 and USP20

The deubiquitinase USP33 has been linked to β-adrenergic receptor recycling, centrosome amplification and cancer cell migration [94,95,96]. Moreover, it plays a central role in the regulation of the GTPase RALB (Ras-like B) [97]. RALB is involved in both innate immune response to viral infection, as well as the induction of autophagy as a cellular response to nutrient availability [98,99]. The decision of which signaling cascade is initiated by RALB depends on its ubiquitination status. Mono-ubiquitinated RALB associates with SEC5(EXOC2), which is a component of the exocyst [100]. This activates the innate immunity signaling kinase TBK1 and, finally, the transcription factor IRF3, which enables the subsequent interferon response. Starvation-induced deubiquitination of RALB by USP33 (Figure 1c) prevents the association with SEC5 and triggers the interaction to another exocyst subunit, EXO84 [97]. Upon activation by RALB, EXO84 assembles with the kinase ULK1, as well as with the PI3K-III complex component Beclin 1, which finally leads to the induction of autophagy [97,101]. Therefore, deubiquitination by USP33 supports autophagy induction.

Recently, an additional possible link to another autophagic process has been uncovered. USP33 and USP20 have been known before to regulate the lysosomal degradation of the β2-adrenergic receptor (β2-AR) [102]. The availability of β2-AR on the plasma membrane surface has to be tightly regulated, and internalization, as well as recycling rates have to be adjusted, because a prolonged downregulation of β2-AR is a hallmark of human heart failure [103].

While the HECT-type E3 ligase Nedd4 mediates the agonist-dependent ubiquitination, internalization and subsequent endocytic targeting of β2-AR to the lysosome [104], USP33 and USP20 deubiquitinate the receptor and prevent its degradation [102] (Figure 1h). Interestingly enough, a recent study demonstrates that the post-endocytic trafficking of ubiquitinated β2-AR involves the fusion of the transport vesicles with autophagosomes before reaching the lysosome [105]. This represents an unusual route, because the endosome-bound β2-AR could reach the lysosome directly via late endosomes/multivesicular bodies. At least in the case of USP20, it was shown that its activity could prevent the uptake of β2-AR in autophagosomes. This occurred in a phosphorylation-dependent manner, because only the non-phosphorylated USP20 was active. Therefore, at least USP20 plays a post-endocytic role in β2-AR trafficking.

Future work may clarify the functional relationship of USP33 and USP20 and if they are always cooperating partners, like in the case of β2-AR, and, therefore, if they are possibly both involved in the regulation of RALB-dependent autophagy.

### 2.4. AMSH

AMSH (associated molecule with the SH3 domain of STAM) is a zinc metallo-deubiquitinase of the JAMM type [106,107]. It participates in the sorting of several cell-surface molecules at late endosomes and has been identified to interact with the ESCRT-III subunits CHMP1A, CHMP1B, CHMP2A and CHMP3 [108,109]. Impairment of AMSH results in severe neuronal damage in mouse models [110]. This effect may not solely be caused by missorting of plasma membrane receptors, because an accumulation of ubiquitinated protein aggregates was observed in these cells [111]. Moreover, the autophagy receptor p62 accumulated at the aggregates, indicating that also the autophagic flux was disturbed [111]. Therefore, the endosomal AMSH also displays a function in autophagy.

In higher eukaryotes, the efficient lysosomal degradation of autophagic cargo often requires the fusion of autophagosomes with late endosomes [112,113]. This formation of so-called amphisomes requires the function of endosomal ESCRT proteins for the delivery of autophagic cargo to lysosomes [8,9,114]. Therefore, the ESCRT-III binding AMSH might play a role in the formation of amphisomes (Figure 1g). The mechanistic role of AMSH in this process is unknown. It might possibly modulate the ubiquitin chains on endosomal cargoes to achieve a better fit for ubiquitin receptors or, alternatively, it might deubiquitinate and stabilize putative factors required for the fusion.

Further evidence for this assumption comes from studies of AMSH1, the orthologous protein in *Arabidopsis thaliana* [115,116]. It interacts with the ESCRT-III subunit VPS2.1 (vacuolar protein sorting 2.1). Similar to known autophagy-mutants of plants, the functional impairment of AMSH1 results in early senescence and hypersensitivity to artificial carbon starvation in the dark. Moreover, the mutants display an accumulation of autophagosomal markers and a reduced amount of autophagic bodies in the vacuole. Thus, AMSH1 and the ESCRT-III-subunit VPS2.1 are both important for autophagic degradation [115,116].

In summary, AMSH is required for the clearance of protein aggregates via autophagy. Furthermore, the AMSH^−/−^ mice provide an animal-model for neurodegenerative diseases, which are commonly characterized by the generation of proteinaceous aggregates.

### 2.5. USP8, USP15, USP30 and USP35

Mutations within the RBR (RING-between-RING)-type E3 ligase Parkin are the most common cause of Parkinson`s disease (PD) [117]. A central role in this context is the function of Parkin to survey mitochondrial quality and integrity. One contribution of Parkin is the modification of the signaling molecule NEMO (NF-κB essential modulator) with linear, non-proteolytic Ub-chains, which contributes to mitochondrial integrity and suppresses apoptosis [118]. The other contribution is the regulation of mitophagy via the ubiquitination of mitochondrial membrane proteins. This type of mitochondrial quality control is executed by the autophagic removal of depolarized mitochondria and their subsequent disposal in the lysosome [119,120,121,122]. Therefore, it is assumed that Parkin-dependent mitochondrial quality control plays a critical role in the protection against Parkinsonism [123,124].

Parkin-mediated mitophagy is initiated by the association of cytosolic Parkin to depolarized mitochondria, which depends on the mitochondrial kinase PINK1 (PTEN-induced putative kinase 1) [125,126]. Parkin ubiquitinates proteins of the outer mitochondrial membrane, as demonstrated for Mitofusin 1, Mitofusin 2 or VDAC1 [119,120,121,122]. The accumulation of ubiquitinated mitochondrial proteins attracts the autophagy-receptor p62 [120,121], which simultaneously binds to ubiquitin and LC3 [127]. More recently, NDP52 and Optineurin were also discussed as mitophagy-receptors [128,129]. Finally, the mitochondria are engulfed by autophagosomes before they are transported to the lysosome [130].

Mitophagy is supported by the deubiquitinase USP8(UBPY) [131] (Figure 1e). Another cell-protective function of USP8 concerns its role in the DNA damage response, where it deubiquitinates BRIT1 (breast cancer susceptibility gene C terminus-repeat inhibitor of human telomerase repeat transcriptase expression 1), resulting in the targeting of BRIT1 to DNA breaks [132]. Moreover, the inherited mutations of USP8 lead to Cushing`s disease [133,134].

In the context of mitophagy, USP8 specifically removes Lys6-linked polyubiquitin chains from Parkin and thereby supports the autophagic degradation of mitochondria [131]. The non-canonical Lys6-chains may stem from an inhibitory auto-ubiquitination of Parkin, which may possibly block the association with interaction partners [135]. Only the deubiquitinated Parkin can efficiently reach the depolarized mitochondria [131].

In the context of its cancer-related function, USP8 was shown to be inhibited by the compounds HBX (Hybrigenetics) 90,397 and 9-ethyloxyimino-9*H*-indeno[1,2-*b*]pyrazine-2,3-dicarbonitrile [136,137,138]. It will be of interest to test whether HBX 90,397 also interferes with the mitophagy-related function of USP8.

Mitophagy can be negatively regulated by the deubiquitinases USP15 [139], USP30 [140,141] and USP35 [141]. These enzymes do not act on Parkin, but deubiquitinate the mitochondrial targets of Parkin. This means that they eliminate the recognition signal for the autophagy-receptors and thereby prevent the uptake into autophagosomes [142].

It has been reported that a knockdown of USP15 can rescue the mitophagy defect of PD patient fibroblasts, which exhibit mutated Parkin and decreased Parkin protein levels. This result suggests that USP15 acts as an antagonist of Parkin and therefore could represent a possible target for a therapeutic strategy for PD patients with reduced Parkin protein levels [139].

A similar correlation has been found for USP30 and Parkin [141,143]. USP30 opposes the occurrence of Parkin-dependent Lys6-, Lys-11- and Lys63-linked polyubiquitin chains on mitochondrial proteins [143]. Furthermore, it has been demonstrated that overexpression of USP30 removes the ubiquitin from mitochondrial targets of Parkin. The reduction of USP30 activity enhances mitophagy in neurons. Moreover, the knockdown of USP30 partially complements the defective mitophagy caused by pathogenic mutations in Parkin and improves mitochondrial integrity [143]. Therefore, the inhibition of USP30 is assumed to be potentially beneficial for Parkinson's disease by promoting mitochondrial clearance and quality control [140,144].

USP35 seems to act via a somewhat distinct mechanism. Even though it also delays Parkin-dependent mitophagy, it does not hamper the association of Parkin with mitochondria. Moreover, it only associates with depolarized mitochondria [141].

Based on these data, it will be an important issue to develop small molecules that act as specific DUB-inhibitors and could potentially be tested in the treatment of Parkinson's disease. It is interesting to note that the diterpenoid derivative 15-oxospiramilactone (S3) has been identified as an inhibitor of USP30 [145]. This was discovered in the context of mitochondrial fusion. The fusion process requires the non-proteolytic ubiquitination of the mitochondrial proteins Mitofusin 1 and Mitofusin 2 by an unknown E3 enzyme. The modification of the Mitofusins is reversed by USP30, thereby regulating mitochondrial morphology [146,147]. This USP30-mediated block can be abolished by the inhibitor S3 [145]. It will be of interest to elucidate if S3 can also abolish the USP30-dependent downregulation of mitophagy in Parkinson’s disease.

Interestingly, Parkin has been shown to be involved in autophagy also in the context of Alzheimer’s disease (AD). With the help of animal AD models, it has been demonstrated that Parkin ubiquitinates amyloid-β, which results in the Beclin 1-dependent autophagic clearance of ubiquitinated Amyloid-β and dysfunctional mitochondria [148,149,150]. The Beclin 1-dependent clearance of intraneuronal amyloid-β has been suggested to oppose extracellular plaque deposition and restore neurotransmitter balance [151,152,153,154].

However, if USP8, USP15, USP30 or USP35 is also involved in the regulation of the AD-related functions of Parkin remains to be elucidated.

Furthermore, the deubiquitinases USP2 [131] and Ataxin-3 [155] have been shown to act on Parkin. However, it is not known if they also have a specific influence on autophagy. In summary, the distinct contributions of USP8, USP15, USP30 and USP35 highlight the functional role of these DUBs in mitophagy and, therefore, possibly also in neurodegenerative diseases.

### 2.6. USP36

The selective autophagic degradation of protein aggregates is called aggrephagy [156,157]. Here, toxic protein aggregates are ubiquitinated and then recognized by autophagy receptors, which results in their engulfment by autophagic membranes and, finally, lysosomal disposal.

The function of the deubiquitinase USP36 has been shown to counteract this mechanism [158] (Figure 1d). The deletion of USP36 of *Drosophila melanogaster* results in an inhibition of larval growth and activated constitutively autophagy in an mTOR-independent manner. The accumulation of ubiquitinated nuclear aggregates, which include histone H2B, as well as of ubiquitinated proteins in the cytosol, was observed. Moreover, it was shown that the p62/Ref(2)P-dependent pathway was affected both in *D. melanogaster*, as well as in human cells [158]. Therefore, USP36 inhibits the selective autophagy of protein aggregates at an early stage by removing the ubiquitin signal.

Another link of USP36 to the autophagy-machinery and cancerogenesis concerns its interplay with c-Myc. The proto-oncogene c-Myc plays a central role in the induction of cell proliferation and is highly regulated by the ubiquitin proteasome system. Aberrant stabilization of c-Myc is associated with several types of human cancers [159]. It has been known before that c-Myc is ubiquitinated by several E3 ligase, including the SKP1-cullin-1-F-box complex, whereas it is deubiquitinated and stabilized by USP28 [160]. Recently, it was demonstrated that the degradation of c-Myc in the nucleolus is antagonized by USP36 [161]. An elevated expression level of USP36 is detected in several types of human breast and lung cancers [162].

The function of c-Myc is antagonized by the PI3K-III complex subunits Beclin 1 and Ambra 1 [163,164]. Especially Ambra 1 is thought to mediate the interaction between the active, phosphorylated c-Myc and its site-specific phosphatase PP2A (protein phosphatase 2A). Because the dephosphorylated c-Myc is prone to ubiquitination and degradation [165], the autophagy-related Ambra 1 acts as a haploinsufficient tumor suppressor gene. However, a direct functional interconnection of USP36-mediated deubiquitination of c-Myc that opposes Beclin 1-/Ambra 1-induced ubiquitination of c-Myc remains to be demonstrated.

USP36 seems to antagonize different autophagy-related processes in a context-dependent manner. It directly deubiquitinates the polyubiquitinated protein aggregates and prevents their recognition by autophagy-receptors and disposal by aggrephagy. Moreover, it principally seems to oppose the function of the PI3K-III signaling complex members Beclin 1 and Ambra 1 concerning the destabilization of the proto-oncogene c-Myc, because it deubiquitinates and thereby stabilizes c-Myc.

### 2.7. SseL

While the eukaryotic cell uses ubiquitination and xenophagy [4] to remove pathogens, it is interesting to note that bacteria themselves can utilize a deubiquitinating enzyme to counteract the cellular autophagy pathway. It has been shown for *Salmonella enterica* serovar Typhimurium that it can produce and secrete the deubiquitinase SseL [166].

S. Typhimurium is a facultative intracellular pathogen, which can cause diseases of the Salmonellosis spectrum [167]. After invasion of the host cell, it resides within a membrane-bound compartment called the Salmonella-containing vacuole (SCV) [168,169,170]. It can translocate SseL and other proteins to the cytosol via the Salmonella pathogenicity island (SPI)-2 type 3 secretion system (T3SS). However, this connection with the cytosol makes it also prone to be recognized by the ubiquitin-system and, therefore, also by the defense mechanisms of the cell. It has been shown that an overactive secretion machinery can partially damage the SCV, which results in its ubiquitination and degradation via autophagy [168,171]. Another difficulty for the bacteria are aggresome-like induced structures (ALIS). These stress-induced protein storage compartments are often formed when the bacteria enter the cell. Normally, ALIS is ubiquitinated and degraded either via the proteasome or via autophagy [172]. Therefore, ALIS formation induces an upregulated presence of autophagy receptors and autophagic flux, which finally harms also the SCVs.

S. Typhimurium counteracts this mechanism by translocating the virulence protein SseL (Salmonella-secreted factor L) (Figure 1f). The deubiquitinase SseL removes the ubiquitin from ALIS and SCVs [173]. As a consequence, the autophagy receptor p62 cannot recognize these structures anymore and the autophagic flux is lowered. This situation favors the intracellular replication of *Salmonella* [173,174].

While earlier work had suggested that SseL protects the bacteria by downregulation of the NF-κB pathway [175], a recent study could not support this concept [176]. Therefore, the current opinion on the virulence mechanism of *Salmonella* is that it facilitates its protection against the cellular defense via the DUB SseL by the downregulation of selective autophagy [173,174].

## 3. Conclusions

Autophagy is a central cellular process required for the degradation of aberrant cytosolic components, like protein aggregates, organelles or intracellular pathogens. In higher eukaryotes, these targets are often primed for their autophagic disposal with the signaling protein ubiquitin. Work in recent years has uncovered the important role of deubiquitinases in this context (Table 1).

Deubiquitination occurs mostly in the early steps of autophagy. The VPS34/Beclin 1-based signaling complexes, which are already active prior to the full formation of the pre-autophagic structures, are regulated in a context-dependent manner by several deubiquitinases. While A20 inhibits PtdIns3P signaling by removing the TRAF6-dependent Lys63-linked chains from Beclin 1, the enzymes USP10 and USP13 prevent PI3K-III complex components from their degradation and, therefore, support autophagy. Interestingly enough, USP10 also stabilizes p53, which, in turn, triggers the degradation of Beclin 1 and VPS34 in order to prevent autophagy. Another example in the context of early signaling events is the deubiquitination of RALB by USP33, which enables the interaction of non-modified RALB with Beclin 1 complexes in order to induce autophagy.

**Table 1:** List of deubiquitinating enzymes involved in autophagy. An overview of the involvement of deubiquitinases in autophagy. Listed are the names (deubiquitinase), enzymatic sub-family (DUB-type), intracellular localization (localization), their role in certain parts of the autophagy-pathways (involvement in autophagy), the known autophagy-relevant target (autophagy-related target), their general effect on the progression of autophagy (effect), the general type of disease they are associated with (disease-type), known specific inhibitors (inhibitors), as well as important citations (references).

Deubiquitinase	DUB-type	Localization	Involvement in Autophagy ^1^	Autophagy-related Target ^1^	Effect on Autophagy	Disease Category ^1,2^	Inhibitor ^3^	References
A20 (TNFAIP3)	OTU	cytosol , lysosome	signaling xenophagy	Beclin 1NDP52	**-** **-**	Inflammation; Cancer?		[57,58,59,60,61,63,65,76]
AMSH	JAMM	endosome	amphisome	endosomal cargo	**+**	Neuro-degeneration		[109,110,111]
SseL	bacterial DUB	cytosol	xenophagy	ALIS, SCV	**-**	Bacterial infection		[173]
UCH-L1	UCH	ER, cytosol, nucleus	?	?	**-**	Neuro-degeneration	LDN91946; Isa- tin O-acyloxime	[53,54,55]
USP8 (UBPY)	USP	cytosol, endosome	mitophagy	Parkin	**+**	Neuro-degeneration?	HBX 90,397; 9-E	[131,136,137,138]
USP10	USP	cytosol, nucleus	signaling signaling	Beclin 1 p53	**+** **-**	Cancer	Spautin 1	[79,82,83,89,91]
USP13	USP	cytosol, nucleus	signaling	Beclin 1	**+**	Cancer	Spautin 1	[79,82,83]
USP15	USP	mitochondrion	mitophagy	mito. proteins	**-**	Neuro-degeneration		[139]
USP20	USP	cytosol, cytoskeleton	amphisome	ß2-AR	**-**	Cancer		[102,105]
USP22	USP	cytosol, nucleus	?	?	**+**	Cancer		[51]
USP30	USP	mitochondrion	mitophagy	mito. proteins	**-**	Neuro-degeneration	15-oxospira-milactone(S3)	[140,141,143,145]
USP33	USP	cytosol, cytoskeleton, Golgi apparatus	signaling amphisome	RalB ß2-AR	**+** **-**	Cancer		[94,95,96,97,102]
USP35	USP	mitochondrion	mitophagy	mito. proteins	**-**	Neuro-degeneration		[141]
USP36	USP	cytosol, nucleus	aggrephagy	prot. aggregate c-Myc	**-**	Cancer, Neuro-degeneration		[158,161,162]

We refer to the manuscript text for more detailed information. ^1^ Please note that this is the autophagy-related information. Certain DUBs may have other targets or are associated with other diseases in a different context. ^2^ Please note that this is only a very general categorization. For instance, in the case of an association with cancers, possibly only certain types of cancers were tested, but not all. Moreover, the information refers to the assumed autophagy-related function of the corresponding DUB. ^3^ Please note, that this is the available information on inhibitors. They are assumed to be specific for the corresponding DUB, but may not be tested in every condition. Moreover, only Spautin 1 has been tested in the context of autophagy, while the other inhibitors were tested in the context of cancer cells. 9-E stands for 9-ethyloxyimino-9*H*-indeno[1,2-*b*]pyrazine-2,3-dicarbonitrile. Symbols:? (unknown); + (pos. effect); - (neg. effect).

Most of the known examples of autophagy-related DUBs target the modified autophagy substrate and cleave off the ubiquitin in order to prevent the recognition by autophagy receptors. This has been described for USP36, which targets ubiquitinated protein aggregates, or the deubiquitinases of mitochondrial proteins, like USP15, USP30 and USP35. Only USP8 supports mitophagy by stabilizing the E3 ligase Parkin. A special variation to this theme represents the bacterial DUB SseL, which is secreted by *Salmonella* bacteria. SseL acts as a virulence factor by removing ubiquitin from bacteria-containing vesicles in order to prevent them from autophagic clearance. The activity of SseL should hide the SCVs and associated ALIS from being recognized by autophagy-receptors. Interestingly enough, the function of the corresponding autophagy-receptor NDP52 has been demonstrated to be dependent on a balanced action of the E3 TRAF6 and the endogenous DUB A20.

The few examples of late acting DUBs in autophagy concern DUBs that are associated with endosomes and that regulate the formation of amphisomes, like AMSH, USP20 and USP33. While USP20 and USP33 antagonize this process for selective cargoes, the activity of AMSH in general is required, probably via the optimal modulation of the ubiquitin chains on the targeted endosomal cargo.

Because it has been established by now that ubiquitination plays a crucial role in the autophagic pathways of eukaryotic cells, the systematic elucidation of the functional role of deubiquitinating enzymes in the regulation of these processes is the next logical step. However, only in recent years have several of the corresponding deubiquitinases been identified and characterized, while for many ubiquitin-dependent autophagy pathways, the counteracting DUB is still unknown. Moreover, the screen for small compounds that act as inhibitors for individual DUBs is of central interest. Taking into account the central role autophagy plays in health and disease, the study of the deubiquitinating enzymes will very likely result in the identification of novel diagnostic tools, therapeutic approaches and potential drug targets.
